# Daily activity locations k-anonymity for the evaluation of disclosure risk of individual GPS datasets

**DOI:** 10.1186/s12942-020-00201-9

**Published:** 2020-03-05

**Authors:** Jue Wang, Mei-Po Kwan

**Affiliations:** 1 Department of Geography, University of Toronto Mississauga, 3359 Mississauga Road, Mississauga, ON L5L 1C6 Canada; 2 Department of Geography and Resource Management, The Chinese University of Hong Kong, Shatin, Hong Kong, China; 3 Institute of Space and Earth Information Science, The Chinese University of Hong Kong, Shatin, Hong Kong, China; 4 Department of Human Geography and Spatial Planning, Utrecht University, 3584 CB Utrecht, The Netherlands

**Keywords:** Geoprivacy, Geomasking, k-anonymity, GPS datasets, Confidential geospatial data

## Abstract

**Background:**

Personal privacy is a significant concern in the era of big data. In the field of health geography, personal health data are collected with geographic location information which may increase disclosure risk and threaten personal geoprivacy. Geomasking is used to protect individuals’ geoprivacy by masking the geographic location information, and spatial k-anonymity is widely used to measure the disclosure risk after geomasking is applied. With the emergence of individual GPS trajectory datasets that contains large volumes of confidential geospatial information, disclosure risk can no longer be comprehensively assessed by the spatial k-anonymity method.

**Methods:**

This study proposes and develops daily activity locations (DAL) k-anonymity as a new method for evaluating the disclosure risk of GPS data. Instead of calculating disclosure risk based on only one geographic location (e.g., home) of an individual, the new DAL k-anonymity is a composite evaluation of disclosure risk based on all activity locations of an individual and the time he/she spends at each location abstracted from GPS datasets. With a simulated individual GPS dataset, we present case studies of applying DAL k-anonymity in various scenarios to investigate its performance. The results of applying DAL k-anonymity are also compared with those obtained with spatial k-anonymity under these scenarios.

**Results:**

The results of this study indicate that DAL k-anonymity provides a better estimation of the disclosure risk than does spatial k-anonymity. In various case-study scenarios of individual GPS data, DAL k-anonymity provides a more effective method for evaluating the disclosure risk by considering the probability of re-identifying an individual’s home and all the other daily activity locations.

**Conclusions:**

This new method provides a quantitative means for understanding the disclosure risk of sharing or publishing GPS data. It also helps shed new light on the development of new geomasking methods for GPS datasets. Ultimately, the findings of this study will help to protect individual geoprivacy while benefiting the research community by promoting and facilitating geospatial data sharing.

## Background

Personal privacy is a significant concern in the era of big data. Data contributors are at the risk of being identified and having their personal privacy violated if their data are not handled properly. With the development of new technology, collecting and analyzing a large volume of individual data is much easier than before. Most personal data are actually being collected without the notice of data contributors as they surf on the Internet and use mobile phone applications. In the field of health geography, personal health data are collected with geographic location information including residential addresses and daily activity locations to evaluate individual environmental exposures [1–8]. With the growing use of geographic information techniques, accurate personal location information can be easily collected and analyzed. While high-accuracy geospatial data facilitate the improvements in health geography studies, personal location information can be easily linked to other digital data sources and thus may help the identification of individuals [9–11]. For health geography researchers who handle personal geospatial data, assuring individual geographic privacy and confidentiality—geoprivacy—is an important topic [12]. Geoprivacy is an emerging topic that attracts much attention from both researchers and the general public [13, 14].

Because of the geoprivacy concern, confidential geospatial data collected by one scholar or institution cannot be easily shared with others even though sharing these data will benefit the research community of health geography and advance science broadly. The existing procedure for sharing confidential data is often burdensome and costly both for researchers who try to make data widely available and for those trying to get access to and use these data. It creates a huge obstacle for data sharing in the research community and a significant waste of resources for data collection. The difficulties of sharing confidential geospatial data also constrain the ability to replicate and reproduce research—a corner-stone of the scientific paradigm [13].

Scholars have invested great effort in how to securely share confidential geospatial data [15–18]. Basically, there are two broad groups of methods—spatial aggregation and geomasking—for protecting geoprivacy and sharing data securely. First, spatial aggregation [19] aggregates individuals or averages their attributes over either administration areas (e.g., census tracts) or pre-defined geographic regions (e.g., uniformed grid). The second group of methods, geomasking—proposed by Armstrong et al. [20]—randomly perturb individuals’ geographic locations to other potential places associated with the original point. There are many ways, such as donut masking [21] and location swapping [22], to manipulate the locations in geospatial data [23–27], and the goal is to use the new locations to substitute for the true locations. With geomasking, individuals’ geoprivacy is protected to some degree while the accuracy of the data is preserved to some extent. It is clear that there is a trade-off between achieving greater geoprivacy using larger geomasking distance to prevent disclosure of individuals’ locational information and achieving higher precision with smaller relocation range to preserve the accuracy of research findings [28, 29]. Through manipulating the maximum relocation distance, a desired trade-off or balance between the degree of geoprivacy and level of accuracy may be achieved [15, 16, 20].

Spatial k-anonymity is widely used to measure the disclosure risk of confidential geospatial data after geomasking [30–32]. It was introduced by Sweeney [33] as a quantitative estimate of the probability of re-identifying a person’s location in a dataset. The value of *k* represents the number of potential locations that could be identified as the true location of an individual so that the probability of being identified by a hacker is at most 1/k [34]. The larger the k, the smaller the chance for a person’s true location to be re-identified. Spatial k-anonymity is designed for geospatial point data (e.g., home locations of individuals). Many studies relied on spatial k-anonymity to evaluate the performance of geomasking methods and understand the degree of disclosure risk of specific geospatial datasets [22, 35]. Spatial k-anonymity can also be used to claim a certain level of privacy protection of an open dataset when actual household locations are masked (e.g., North Carolina E911 database [16] and emergency department visits for respiratory illness [26]), and be “used in Location-based Services to protect privacy, by hiding the association of a specific query with a specific user” [36–38]. To date, there is no standard for the level of desirable or required confidentiality before publishing individual-level spatial data, but achieving a high level of spatial k-anonymity could be a general guideline for researchers [35].

With the recent advances in data collection technology, portable GPS trackers are widely used by health geographers and scientists in other fields to understand human movement and behavior. Collected by portable GPS, individual tracking data contain high-resolution geographic location information of personal daily activities. Instead of single geographic locations for each person in conventional geospatial data, millions of geographic locations are recorded along each person’s movement trajectories in individual GPS trajectory data. Individual GPS trajectory data contain rich information that indicates the locations and time of individuals’ routine daily activities and trips. Compared to the geospatial data that include only people’s home location, the rich spatiotemporal information in GPS data, where detailed individual GPS logs are included, can be used as a strong personal identifier [39], which significantly increases the risk of re-identification. Spatial k-anonymity may work well for conventional health-related geospatial datasets in which only one geographic location is recorded for each person, but the disclosure risk of individual GPS data cannot be assessed by the spatial k-anonymity method since there are many daily activity locations (e.g., home, workplaces, and shopping places) that can be easily discovered from a GPS dataset.

To address some of the limitations of the conventional k-anonymity method. Nergiz et al. [40] proposed trip k-anonymity as a measure of the geoprivacy of an individual’s GPS trajectories. Trip k-anonymity is “considered individually by trip and requires that each be attributable to at least one other person in the data set” [39]. This trip k-anonymity method provides a new tool for researchers to analyze the disclosure risk of GPS datasets. It considers, however, only the similarity among trip trajectories in the same dataset and is calculated as the number of other trip trajectories in a dataset that one trip trajectory can be indistinguishable from [41]. An individual’s GPS trajectories itself, if combined with other contextual data, can be easily used to identify the persons’ activity locations and schedules. With these detailed activity locations abstracted from the GPS trajectories, this individual could be re-identified regardless of other trajectories in the dataset. Therefore, the trip k-anonymity method may not be able to comprehensively measure the disclosure risk of individual GPS data.

This study develops and proposes a new k-anonymity method, as a supplement to spatial k-anonymity, for evaluating the disclosure risk of individual GPS data. Instead of calculating disclosure risk based on one geographic location (e.g., home) of an individual, the new k-anonymity is a composite evaluation of disclosure risk based on all activity locations and the time spent at each location abstracted from the individual’s GPS data. Because this new method assesses disclosure risk from a perspective of daily activity locations, we call it daily activity locations k-anonymity, or DAL k-anonymity. This new method provides a quantitative means to assess and understand the disclosure risk of sharing or publishing individual GPS data. It can also evaluate the effectiveness of new geomasking methods in protecting geoprivacy when these methods are applied to GPS data. Ultimately, the findings of this study will help to protect individual geoprivacy and benefit the research community by facilitating and promoting geospatial data sharing.

## Methods

### Spatial k-anonymity

Spatial k-anonymity is the most widely embraced approach to evaluating the degree of geoprivacy achieved subsequent to the application of specific geomasking technique [30–32, 35]. It is originated from the concept of k-anonymity and was first introduced by Sweeney [33] to quantitatively assess the probability of identifying an individual record from a group of individuals in tabular data. K-anonymity is defined as the number of individuals (the value of k) sharing similar attributes (e.g., gender and ethnicity) so that a particular individual cannot be distinguished in the dataset [16]. In the last few decades, health geographers have started to use geospatial data to investigate health-related issues. Different from tabular data, geospatial data contain individual information regarding geographic locations (e.g., residential location). Traditional k-anonymity was extended to spatial k-anonymity to consider the disclosure risk of geographic identifiers. Spatial k-anonymity estimates the probability of an individual record being re-identified considering the possibility of reverse geocoding and the number of individuals sharing similar geographic identifiers.

**Fig. 1:**
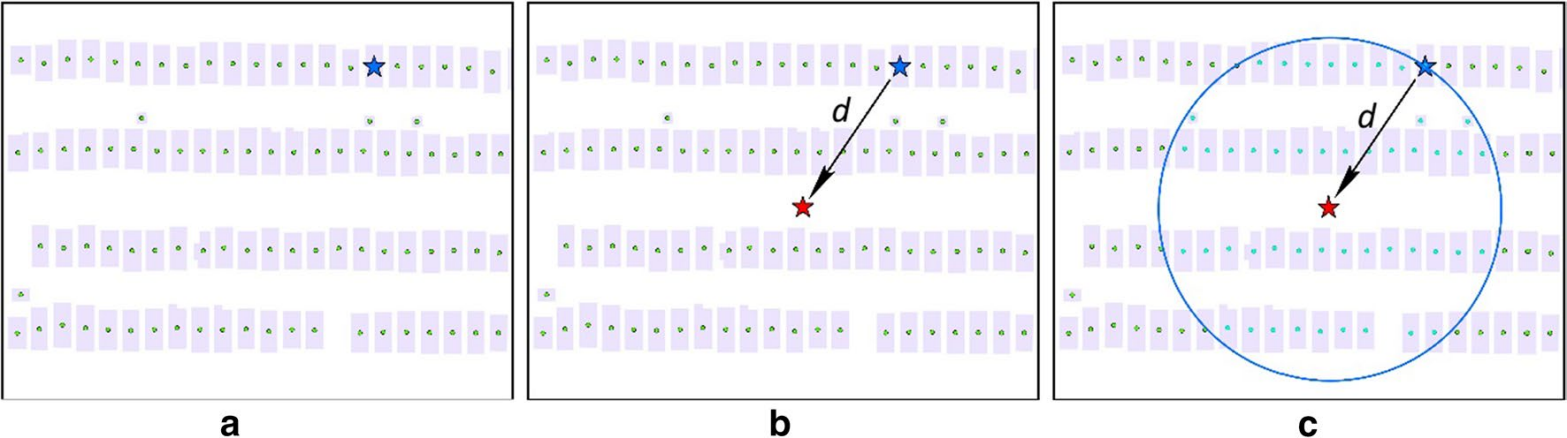
An example of the calculation of spatial k-anonymity. **a** The blue star represents the person’s original location, and the green dots represent the potential residential locations around the person; **b** the person’s masked location is calculated by a geomasking technique and represented as a red star, and the masking distance is *d*; **c** a buffer with a radius of *d* is created around the masked location, and the number of potential residential locations (highlighted green dots) in the buffer area, including the person’s original location, is the value of k for spatial k-anonymity

According to Ghinita et al. [34], the core concept of spatial k-anonymity is that a person’s geographic location is re-located (by a geomasking technique) within an anonymizing spatial region where there are k-1 other potential individuals so that a hacker can re-identify the person in question with a probability of at most 1/k. Thus, spatial k-anonymity is estimated by counting the number of potential individuals in the anonymizing spatial region. One widely used approach to calculating the k is to find the number of neighbors that are closer to the masked location than the distance between the masked and the original locations [16, 23, 35]. Figure 1 illustrates how k-anonymity is determined. The person’s location to be masked is shown as a blue star in the figure. The person’s masked location is obtained by applying a geomasking technique and as a result, the person is relocated to a new location (the red star). The distance between the original location and the new location is the masking distance (*d*). A buffer is then generated centering at the new masked location with a radius of *d*. The k-anonymity value is the number of all potential residential locations inside this buffer area including the original location (the blue star). The value of 1/k is thus a measure of the disclosure risk of the person in question (the probability of being re-identified) after geomasking. The smaller the 1/k, the more difficult it is to re-identify the person’s real location.

### Daily activity locations (DAL) k-anonymity for individual GPS data

Spatial k-anonymity works well for conventional health-related geospatial datasets in which only one geographic location is recorded for each person, yet it has considerable limitations when dealing with individual GPS datasets. Instead of one geographic location that may disclose an individual’s identity, there are many daily activity locations (e.g., home, workplaces, and shopping places) that can be easily discovered from a GPS dataset, and this information can provide rich geographic information for hackers to re-identify a person. Because most people spend only a limited amount of time at their home and spend considerable time away from home [42, 43], it is important to comprehensively consider the disclosure risk associated with all daily activity locations. Therefore, we propose the notion of DAL k-anonymity where the assessment of disclosure risk when using GPS data considers all daily activity locations, and the calculation of DAL k-anonymity is a comprehensive evaluation of the probability of re-identifying these daily activity locations.

DAL k-anonymity is implemented by first abstracting all daily activity locations ($A$) where a specific individual spends at least 20 min daily on average [44]. A kernel-based algorithm [45] is used to detect these activity locations and the time spent at each location. Second, the daily accumulated duration spent at each of these daily activity locations ($T_{A}$) is calculated based on the GPS records (see [45] for the details of calculation). Home location ($A_{h}$) is distinguished from other daily activity locations ($A_{i}$), based on the method in another study [46], by identifying the location where the individual spends a significant amount of time at night. Specifically, we identify an individual’s home location as the location where he/she spends more than 6 h daily and this duration also includes the time of 3 am when most people are sleeping at home. We understand that this approach may not be effective in some low-income areas of the U.S. or other countries where many people work night shifts and the locations where they spend 6 h that also include 3 am are actually work locations. However, our criteria are chosen for the purpose of illustrating the proposed method. Researchers can use or add other appropriate criteria to help identify an individual’s home location accurately (e.g., using the land use of the location to determine whether it is in a residential area). Next, the probabilities of re-identifying home location ($P(A_{h} )$) and other activity locations ($P(A_{i} )$) after geomasking are calculated respectively based on the conventional spatial k-anonymity method. Finally, the probability of identifying the person in question ($P(S)$) is assessed with the following Eqs: 1, 2:

1 $$P\left( S \right) = \mathop \sum \limits_{i = 1}^{n} P\left( {S|A_{i} } \right)P\left( {A_{i} } \right) \times \left( {1 - P\left( {A_{h} } \right)} \right) + P(S|A_{h} )P\left( {A_{h} } \right)$$

2 $$P\left( {S|A_{i} } \right) = \frac{{T_{{A_{i} }} }}{24}$$

where $P\left( {S |A_{i} } \right)$ is the probability of identifying the individual if location $A_{i}$ is re-identified. It is calculated as the weight of the daily time ($T_{{A_{i} }}$) the individual spends at location $A_{i}$ over 24 h. We assume that the longer time the person spends at one activity location, the more important that location is, and thus the higher the probability of identifying the person if this location is re-identified. $P(S|A_{h} )$ is the probability of identifying the individual if his/her home location is re-identified. We assume $P\left( {S |A_{h} } \right)$ equals 100% considering the person is identified if his/her home location is found. Different from other out-of-home daily activity locations (e.g., shopping), the home location is still the one that provides much critical information for identifying an individual and thus has to be considered differently. Because there are normally many other different people conducting the same activity (e.g., grocery shopping) simultaneously at other types of activity locations (e.g., supermarkets), identifying the location of a person’s out-of-home daily activities does not directly lead to the identification of the person although the disclosure risk is increased. But the person is identified in most cases if his/her home location is disclosed. This is also the theoretical foundation of the conventional spatial k-anonymity method, which tries to mask the individual’s true home location in a spatial database to protect privacy.

**Fig. 2:**
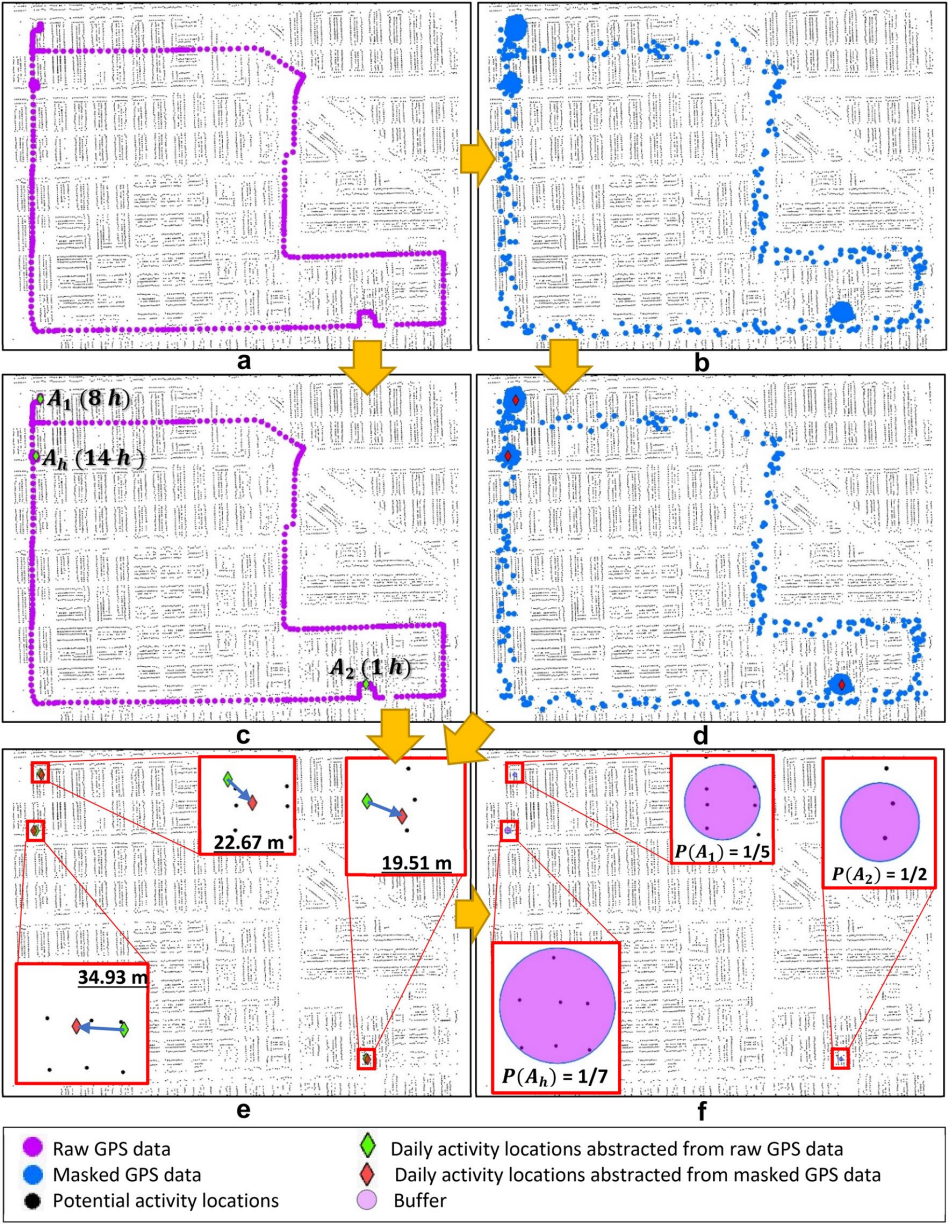
A heuristic example illustrating the calculation of DAL k-anonymity. **a** The simulated raw GPS tracking data; **b** geomasked GPS tracking data; **c** activity locations detected from the raw GPS tracking data; **d** activity locations detected from the geomasked GPS tracking data; **e** the distance between the activity locations detected from the raw GPS tracking data and the geomasked GPS data; **f** the respective probability of re-identifying the different activity locations

The proposed DAL k-anonymity method is further illustrated in Fig. 2 with a heuristic example. Figure 2a shows a simulated GPS dataset tracking an individual for 24 h at a time interval of 1 min. The simulated GPS trajectory was created by the following steps using ArcGIS Pro 2.4. (1) One community in the city of Chicago (downtown area was excluded) was selected as the study area; (2) The centroids of building footprints in the selected community were extracted as the potential activity locations; (3) Three activity locations were randomly selected from these potential activity locations; (4) Each of these three activity locations was randomly assigned as the home, work, and grocery shopping location of a fictitious individual; (5) GPS records were manually created at these locations with a time interval of 1 min specifically according to the assumption that the individual spends 14 h at home, 8 h at work, and 1 h at the grocery shopping location. (6) ArcGIS Network Analysis tool was used to generate the shortest paths among these locations and GPS records were manually generated along these paths. In the subfigures of Fig. 2, the purple dots represent the GPS records while the tiny greyish dots represent all the potential activity locations of the study area. The simulated GPS tracking records are masked with the geomasking methods of random perturbation, and the geomasked GPS records are shown as blue dots in Fig. 2b. Random perturbation, firstly proposed by Armstrong et al. [20], is one of the most widely used geomasking methods [47]. The method displaces each point in a dataset to a random location within a buffer area centered at the original point [20]. The radius of the buffer is typically defined by scholars based on the characteristics of the specific research area (e.g., the population density) [15]. The simulated GPS trajectory used in this study is created in the setting of an urban area in the U.S., so the maximum buffer distance is defined as 200 m, which is adopted in a previous study [22] that implemented geomasking in a U.S. city, for the purpose of illustrating the proposed DAL k-anonymity method.

Thierry et al.’s [45] kernel-based algorithm is implemented to detect activity locations from the raw GPS records. This activity place detection algorithm can accurately discover daily activity locations and the time spent at each location from an individual GPS dataset. With the help of the ArcGIS-based tool provided by Thierry et al. [45], there are three activity locations detected and shown as the green diamonds in Fig. 2c. $A_{h}$ is the detected home location and the person spends 14 h here which include 3 am. $A_{1}$ and $A_{2}$ are two other daily activity locations where the person spends 8 h and 1 h respectively. With a similar analytical process, the three activity locations ($A_{h}$, $A_{1}$, and $A_{2}$) are also detected from the geomasked GPS records (red diamonds in Fig. 2d). Because the geomasking relocated the raw GPS records, these three activity locations detected from the geomasked GPS records are relocated from the ones detected from the raw GPS records. Figure 2e indicates the differences in the distance between these activity location pairs. Centered at each of the activity locations detected from the geomasked GPS records, a buffer is created with a radius as the difference in distance to estimate the probability of re-identifying the different activity locations separately (shown in Fig. 2f). The probability of re-identification is calculated as the disclosure risk based on the conventional k-anonymity method (the number of all potential activity locations inside the buffer area). For example, there are seven potential activity locations inside the buffer area of the detected home location, so the probability of re-identifying the person’s exact home location ($P(A_{h} )$) with the geomasked GPS records is 1/7.

Table 1 lists the detected daily activity locations and their attributes that were used for the calculation of DAL k-anonymity. $P\left( {S |A_{i} } \right)$ is the probability of identifying the person if location $A_{i}$ is re-identified. It is calculated as the weight of time ($T_{{A_{i} }}$) the individual spends at location $A_{i}$ over 24 h. For example, the individual spends 8 h at activity location $A_{1}$, so the probability of identifying the person if location $A_{1}$ is re-identified $P\left( {S |A_{h} } \right) = 8/24$. We assume that the individual is identified if his/her home location is found, so $P\left( {S |A_{h} } \right) = 100\%$. With the attributes calculated at each of these activity locations, the proposed DAL k-anonymity can be calculated for this heuristic example based on Eqs. (1) and (2) as follows:

$$\begin{aligned} P\left( S \right) & = \mathop \sum \limits_{i = 1}^{n} P\left( {S |A_{i} } \right)P\left( {A_{i} } \right) \times \left( {1 - P\left( {A_{h} } \right)} \right) + P\left( {S |A_{h} } \right)P\left( {A_{h} } \right) \\ = \left( {\frac{8}{24} \times \frac{1}{5} + \frac{1}{24} \times \frac{1}{2}} \right) \times \left( {1 - \frac{1}{7}} \right) + 1 \times \frac{1}{7} = 21.79\% . \\ \end{aligned}$$

**Table 1:** Detected daily activity locations and their attributes

Daily activity location	Daily duration ($T_{{A_{i} }}$ in hours)	Whether covered 3 am	Location feature	$P\left( {S|A_{i} } \right)$	$P\left( {A_{i} } \right)$
$A_{h}$	14	Yes	Home	100%	$\frac{1}{7}$
$A_{1}$	8	No	Other location	$\frac{8}{24}$	$\frac{1}{5}$
$A_{2}$	1	No	Other location	$\frac{1}{24}$	$\frac{1}{2}$
Travel	1	No	–	–	–

Based on this calculation of DAL k-anonymity, the disclosure risk of the person’s identity after applying the geomasking on this individual’s GPS tracking data is 21.79%. The $P\left( {S |A_{i} } \right), P\left( {A_{i} } \right), P\left( {S |A_{h} } \right), {\text{and }}P(A_{h} )$ values are abstracted by using the ArcGIS Pro tools (including buffer, intersection, distance measurement, and so on), while the DAL k-anonymity value is calculated manually with the help of Excel.^1^

### Case studies in various scenarios

We present case studies of applying DAL k-anonymity in various scenarios to investigate its performance. The results of applying DAL k-anonymity are also compared with those obtained with spatial k-anonymity under these scenarios. In different case-study scenarios, the characteristics of the individual’s daily activities are manipulated to simulate various daily activity patterns realistically. Table 2 lists these case study scenarios and the characteristics of the individual’s daily activities.

**Table 2:** The five case-study scenarios and the characteristics of the individual’s daily activities in each scenario

Scenario	Travel	Home location		Location A		Location B	
	Time (h)	Time (h)	Number of potential activity locations	Time (h)	Number of potential activity locations	Time (h)	Number of potential activity locations
S1	1	14	1–50	8	5	1	2
S2	1.80–0	6–24	7	14.40–0	5	1.80–0	2
S3	1	14	7	8	1–50	1	1–50
S4	1	14	7	8.57–0.43	5	0.43–8.57	2
S5	1	10	7	The numbers of other activity locations are changed from 1 to 10, and the time spent at these locations are evenly distributed and ranges from 1.3 to 13 h. The numbers of potential activity locations around other activity locations are all set to 5			
S6	1–10	14	7	8–0	5	1–0	2

Scenario 1 (S1): various number of potential activity locations around home. One person spends 14 h daily at home and performs other daily activities at two different locations (A and B). The person spends 8 h at location A and 1 h at location B, while the travel time among these locations is 1 h. The numbers of potential activity locations around locations A and B are 5 and 2 respectively. In this case-study scenario, we keep all these characteristics of the individual’s daily activities fixed and manipulate the number of potential activity locations around the home location from 1 to 50. We will be able to see how DAL k-anonymity varies with the changing numbers of potential activity locations around the home location, and how the results are different from spatial k-anonymity.

Scenario 2 (S2): various time spent at home. One person undertakes daily activities at two different locations (A and B) rather than home. The numbers of potential activity locations around locations A and B and home are 5, 2, and 7 respectively. We keep the above-mentioned characteristics of the individual’s daily activities fixed and manipulate the time the person spends at home from 6 to 24 h. Since the available time spent at other activity locations depends on the time spent at home, we also change the time spent at locations A and B and on travel accordingly so that the time spent on these activities are 8/10, 1/10, and 1/10 of the daily time spent out of home. In other words, the more time spent at home, the less time spent at other activity locations, but the total time would be still 24 h. We will be able to see how DAL k-anonymity varies with the changing time spent at home, and how the results are different from spatial k-anonymity.

Scenario 3 (S3): various number of potential activity locations around other activity locations. One person spends 14 h daily at home and undertakes other daily activities at two different locations (A and B). The person spends 8 h at location A and 1 h at location B, while the travel time among these locations is 1 h. The number of potential activity locations around the home is 7. In this scenario, we keep all these characteristics of the individual’s daily activities fixed and manipulate both the number of potential activity locations around locations A and B from 1 to 50. We will be able to see how DAL k-anonymity varies with the changing numbers of potential activity locations around other activity locations, and how the results are different from spatial k-anonymity.

Scenario 4 (S4): various time spent at other activity locations. One person spends 14 h daily at home and performs other daily activities at two different locations (A and B). The person spends 1 h on travel among these locations. The numbers of potential activity locations around locations A and B and home are 5, 2, and 7 respectively. We keep all these characteristics of the individual’s daily activities constant and manipulate the time spent at both locations A and B. Since the time spent at home and travel is fixed at 15 h, the time the person could spend at locations A and B are 9 h in total. We change the ratio of the time spent at locations A and B from 20:1 to 1:20 while keeping the total time as 9 h. We will be able to see how DAL k-anonymity varies with the changing time spent at other activity locations, and how the results are different from spatial k-anonymity.

Scenario 5 (S5): various number of other activity locations. One person spends 10 h daily at home and spends 1 h for travel among different activity locations. The number of potential activity locations around the home is 7. In this scenario, we change the number of other activity locations from 1 to 10, and the time spent at these locations is evenly distributed ranging from 1.3 to 13 h. The number of potential activity locations around other activity locations is all set to 5. We will be able to see how DAL k-anonymity varies with the changing numbers of other activity locations, and how the results are different from spatial k-anonymity.

Scenario 6 (S6): various time spent on travel. One person spends 14 h daily at home and conducts other daily activities at two different locations (A and B). The numbers of potential activity locations around locations A and B are 5 and 2 respectively. We keep the above-mentioned characteristics of the individual’s daily activities unchanged and manipulate the time the person spends on travel from 1 to 10 h. Since the available time spent at other activity locations depends on the time spent on travel, we also change the time durations spent at locations A and B accordingly so that the time spent at these locations are 8/9 and 1/9 of the available daily time. We will be able to see how DAL k-anonymity varies with the changing time spent on travel, and how the results are different from spatial k-anonymity.

## Results

### Various number of potential activity locations around home

**Fig. 3:**
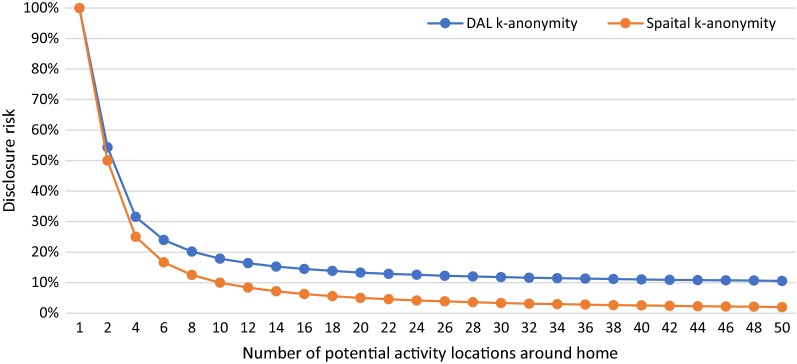
DAL k-anonymity (blue line) and spatial k-anonymity (orange line) with the changing numbers of potential activity locations around home location from 1 to 50

In S1, we manipulate the number of potential activity locations around the home from 1 to 50 to see how DAL k-anonymity varies with changing numbers of potential activity locations around home ($N_{ph}$) and how the results are different from spatial k-anonymity. Figure 3 shows the results of DAL k-anonymity (blue line) and spatial k-anonymity (orange line) in various number of potential activity locations around home. When $N_{ph} = 1$, the results of DAL k-anonymity and spatial k-anonymity are both 100%. With the increase of $N_{ph}$, the disclosure risk of the individual decreases for both methods. However, the value of spatial k-anonymity decreases faster than DAL k-anonymity and becomes infinitely close to zero, while the value of DAL k-anonymity is infinitely close to a value (8.75% in this scenario) that is determined by the number of other activity locations and the number of potential activity locations around these locations.

### Various time spent at home

**Fig. 4:**
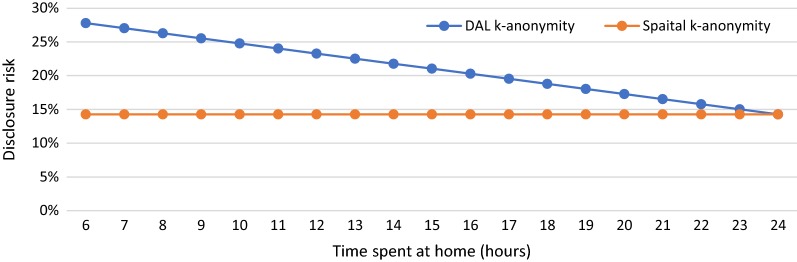
DAL k-anonymity (blue line) and spatial k-anonymity (orange line) with the changing time the person spends at home from 6 to 24 h

In S2, we manipulate the time the person spends at home ($T_{h}$) from 6 to 24 h to see how DAL k-anonymity varies with the changing time spent at home and how the results are different from spatial k-anonymity. Figure 4 illustrates the results of DAL k-anonymity (blue line) and spatial k-anonymity (orange line) for various time durations the person spends at home. It can be seen from the figure that the value of spatial k-anonymity remains unchanged at 14.29% no matter how much time the person spends at home. However, the value of DAL k-anonymity decreases linearly from 27.79 to 14.29% with an increase in the time the person spends at home. When $T_{h} = 24$, which is an extreme condition that the person spends all his/her time at home, the value of DAL k-anonymity equals to that of spatial k-anonymity.

### Various number of potential activity locations around other activity locations

**Fig. 5:**
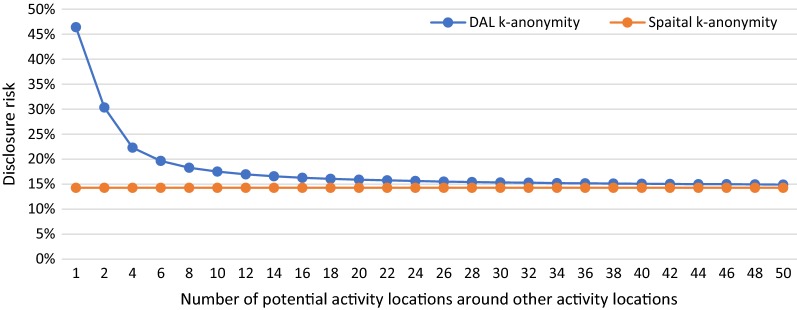
DAL k-anonymity (blue line) and spatial k-anonymity (orange line) for various numbers of potential activity locations around other activity locations from 1 to 50

The numbers of potential activity locations around other activity locations (except home) from 1 to 50 are manipulated in S3. We investigate how DAL k-anonymity varies with the changing numbers of potential activity locations ($N_{pi}$) around other activity locations and how the results are different from spatial k-anonymity. For the purpose of illustration and simplicity of calculation, we assume there are two other activity locations (A and B) and keeps the numbers of potential activity locations around both locations A and B the same. Figure 5 indicates the results of DAL k-anonymity (blue line) and spatial k-anonymity (orange line) for various numbers of potential activity locations around both locations A and B. As shown in the figure, the value of spatial k-anonymity remains the same (14.29%) with changing numbers of potential activity locations around other activity locations from 1 to 50, while the value of DAL k-anonymity decreases from 46.43 to 14.93% and becomes infinitely close to the value of spatial k-anonymity (14.29%) as $N_{pi}$ keeps increasing.

### Various time spent at other activity locations

**Fig. 6:**
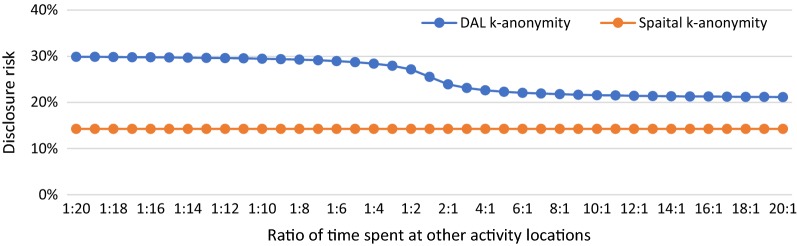
DAL k-anonymity (blue line) and spatial k-anonymity (orange line) for various ratios of time spent at other activity locations

In S4, we manipulate the ratio of the time spent at other activity locations to see how DAL k-anonymity varies with variations of the time spent at other activity locations and how the results are different from spatial k-anonymity. For the purpose of illustration and simplicity of calculation, we assume there are two other activity locations (A and B) and change the ratio of the time spent at locations A and B from 20:1 to 1:20 while keeping the total time as 9 h. It can be seen from Fig. 6 that the value of spatial k-anonymity remains constant as 14.29% with the changing ratio of time spent at other activity locations. For the DAL k-anonymity, the value of DAL k-anonymity changes along with the ratio of time spent but is always higher than the value of spatial k-anonymity.

### Various numbers of other activity locations

**Fig. 7:**
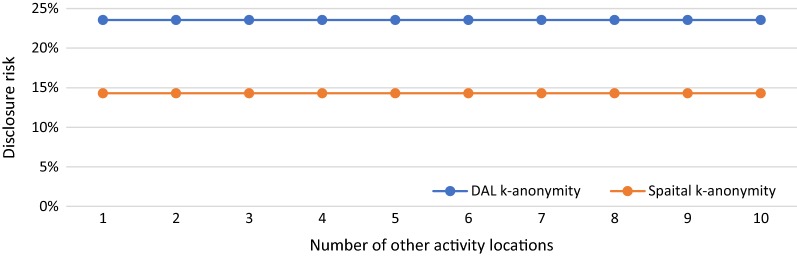
DAL k-anonymity (blue line) and spatial k-anonymity (orange line) in various number of other activity locations

In S5, we manipulate the number of other activity locations from 1 to 10 with a total time of 9 h to see how DAL k-anonymity varies with the changing numbers of other activity locations and how the results are different from spatial k-anonymity. The time durations spent at these locations are evenly distributed and range from 1.3 to 13 h and the numbers of potential activity locations around other activity locations are all set to 5. Figure 7 illustrates the results of DAL k-anonymity (blue line) and spatial k-anonymity (orange line) for various numbers of other activity locations. We can see that both DAL k-anonymity and spatial k-anonymity do not change with various numbers of other activity locations. The value of DAL k-anonymity remains at 23.57%, which is higher than the value of spatial k-anonymity (14.29%).

### Various time spent on travel

**Fig. 8:**
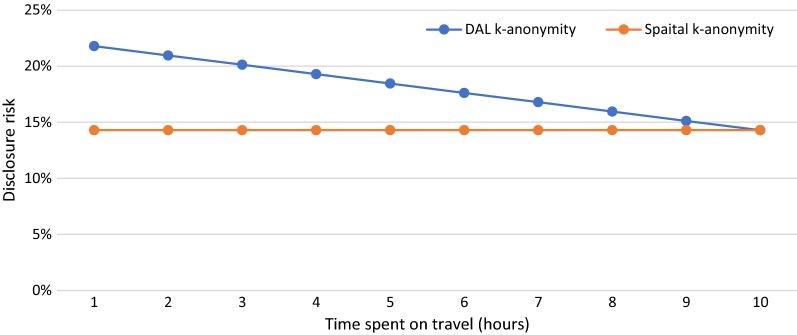
DAL k-anonymity (blue line) and spatial k-anonymity (orange line) for various time the person spends on travel

In S6, we manipulate the time the person spends on travel ($T_{t}$) from 1 to 10 h to see how DAL k-anonymity varies with the changing time spent on travel and how the results are different from spatial k-anonymity. Since the available time spent at other activity locations are dependent on the time spent on travel, we also change the time spent at other activity location accordingly. Figure 8 shows the results of DAL k-anonymity (blue line) and spatial k-anonymity (orange line) for various time the person spends on travel. It can be seen from the figure that the value of DAL k-anonymity decreases from 21.79% with the increase of time spent on travel, while spatial k-anonymity remains unchanged at a value of 14.29%. Since the time spent at home is 14 h in this scenario, when travel time increases to 10 h, there is no time for other activity locations and the value of DAL k-anonymity becomes equal to the value of spatial k-anonymity.

## Discussion

The results of the six case-study scenarios indicate that the value of DAL k-anonymity is always larger or equal to that of spatial k-anonymity. In other words, the disclosure risk calculated by DAL k-anonymity is higher or equal to that calculated by spatial k-anonymity. Spatial k-anonymity only considers the disclosure risk of re-identifying an individual’s home and ignores all the other daily activity locations. The method works well for conventional health-related geospatial datasets that contain only people’s home location. In their daily lives, people undertake different types of activities at different times and locations. And this information can be abstracted from GPS datasets. Thus, ignoring the disclosure risk associated with all these daily activity locations underestimates the overall disclosure risk of GPS data. For DAL k-anonymity, all the daily activity locations including home are considered in the calculation of the disclosure risk. The identification of a persons’ other daily activity location increases the chance for the person to be re-identified. The more time an individual spends at one daily activity location, the more important this activity location is for the individual and the higher the contribution of this location to the overall disclosure risk. Because of the comprehensive evaluation of the disclosure risks at all daily activity locations, the disclosure risk calculated by DAL k-anonymity is normally higher than that obtained with spatial k-anonymity. Only at several extreme conditions (e.g., when $T_{h} = 24$ in S2 and when $T_{t} = 10$ in S6), the DAL k-anonymity equals to spatial k-anonymity. These extreme conditions share the same characteristics that there is no other daily activity location and the individual spends all his/her time at home. Because there is no other daily activity location to increase the chance of being re-identified, it is reasonable that DAL k-anonymity equals to spatial k-anonymity under these circumstances.

The results of this study indicate that DAL k-anonymity provides a better estimation of the disclosure risk than does spatial k-anonymity in many ways. When manipulating the number of potential activity locations around home in S1, we can see from the results that the disclosure risk of home is the most important factor for DAL k-anonymity. If the $N_{ph} = 1$ (the disclosure risk of home is 100%), the individual is re-identified. With the increase of $N_{ph}$ (the disclosure risk of home is decreasing), the disclosure risk of the individual decreases dramatically. However, other activity locations still contribute to the overall disclosure risk and so DAL k-anonymity becomes infinitely close to a value determined by the disclosure risk of other activity locations. Because spatial k-anonymity only considers the disclosure risk of home, so the overall disclosure risk of the individual calculated with spatial k-anonymity decreases with the increase of $N_{ph}$ and becomes infinitely close to zero. For S2 to S6, because spatial k-anonymity is only calculated based on the number of potential activity locations around home, it remains unchanged no matter how we manipulate the characteristics of the individual’s daily activity patterns. While DAL k-anonymity adjusted according to the various characteristics of the individual’s daily activity patterns. As can be concluded from the results of S2 and S6, the less time the person spends at home and travel, the more time he/she can spend at other activity locations and thus the more important these other activity locations are in the re-identification of the individual. Therefore, the disclosure risks calculated with DAL k-anonymity increases with the decrease of time spent at home and travel.

From the results of S3 and S4, it is clear that DAL k-anonymity considers the time spent at other activity locations and the number of potential activity locations around other activity locations as the weight to calculate the overall disclosure risk. The more time the person spends at one activity location and a smaller number of potential activity locations around that location, the more contribution of this location to the overall disclosure risk. For example, in S4, the larger ratio indicates more time spent at location A compared to B and more contribution location A to the overall disclosure risk than location B. Therefore, the overall disclosure risk decreases since the number of potential activity locations around A is more than the ones around B and the disclosure risk of re-identifying location A is lower than B. It is interesting to note that the findings of S5 indicate that DAL k-anonymity remains unchanged with changing numbers of other activity locations as long as the total time spent at all these locations are the same. It is reasonable that more activity locations decrease the time spent at each of these locations and thus decrease the contribution of each location to the overall disclosure risk. Although DAL k-anonymity remains unchanged in S5, it is still higher than spatial k-anonymity because of the consideration of the disclosure risks of all these activity locations.

The proposed DAL k-anonymity comprehensively assesses the probability of re-identifying a person from individual GPS data. Compared to conventional spatial k-anonymity, DAL k-anonymity considers the disclosure risk not only of the home location but also of all other places an individual visits and perform daily activities. Further, the method also considers the average daily time spent at each activity location to distinguish the importance of each location for re-identifying a person. The comprehensive assessment of the disclosure risk of GPS data using DAL k-anonymity performs better than spatial k-anonymity. Therefore, DAL k-anonymity is a more suitable tool for assessing the disclosure risk of GPS datasets and evaluating the performance of different geomasking methods on GPS datasets. In addition, the proposed DAL k-anonymity may provide support for developing new geomasking methods that can effectively mask GPS data to protect personal geoprivacy.

Although the proposed method extends spatial k-anonymity and performs better than conventional spatial k-anonymity when evaluating the disclosure risk of individual GPS datasets, there are some limitations that should be addressed in future research. First, the new method only tested and evaluated a limited number of case-study scenarios. More case studies with different characteristics of individuals’ daily activities may be needed to further evaluate the performance of the proposed DAL k-anonymity method. In addition, the kernel-based algorithm used in this study to abstract daily activity locations may not be accurate enough for discovering all daily activity locations as well as the time spent at each location. Other algorithms for abstracting daily activity locations from GPS datasets are worth investigating for future research. Moreover, using simulated data, this study is a pilot exploration of the method for evaluating the disclosure risk of GPS data. However, more studies using real-world GPS datasets at different locations and in different geographic contexts are needed to further investigate the merits and shortcomings of DAL k-anonymity. Further, this research does not consider the number of people undertaking activities simultaneously at the same activity location due to the feasibility and limitation of data. For example, if a person exercises in a gym, it is possible that there are many other persons performing the same activity at the same location. In future studies, it would be helpful to investigate whether this is an important factor when considering the disclosure risk at people’s daily activity locations. Lastly, as shown by the results of this study, the existing geomasking methods developed based on conventional geospatial data may not be effective for protecting people’s geoprivacy when using GPS data. The development of new geomasking methods that can more effectively mask GPS data is thus an important topic for future research.

## Conclusions

In this article, we proposed the DAL k-anonymity method that comprehensively assesses the probability of re-identifying individuals from GPS datasets. DAL k-anonymity considers the disclosure risk of an individual’s all daily activity locations discovered from GPS records. Different from the spatial k-anonymity method that calculates disclosure risk based only on people’s home location, DAL k-anonymity is a composite evaluation of disclosure risk based on all activity locations of individuals and the time they spend at each location. Comparing the results obtained with spatial k-anonymity under various case-study scenarios, the study indicates that DAL k-anonymity provides a more effective method for evaluating the disclosure risk when using individual GPS data. This new method provides a quantitative means to understand the disclosure risk of sharing or publishing GPS data. It also helps to shed new light on the development of new geomasking methods for GPS dataset. Ultimately, the findings of this study will help to protect individual geoprivacy while benefiting the research community through promoting and facilitating geospatial data sharing.

## Data Availability

The dataset generated and analyzed in the current study is not publicly available. It will be available from the authors on reasonable request after the project is completed.

## Abbreviation

DAL k-anonymity: daily activity locations k-anonymity

## Footnotes

1 The authors’ research team is currently developing an ArcGIS-based tool for the DAL k-anonymity analysis. The authors will be happy to share the simulated GPS trajectory dataset and tools with interested researchers after finishing this work.
